# Reflections throughout the COVID-19 Lockdown: What Do I Need for Successful Learning of Engineering?

**DOI:** 10.3390/ijerph182111527

**Published:** 2021-11-02

**Authors:** Víctor Revilla-Cuesta, Marta Skaf, Milagros Navarro-González, Vanesa Ortega-López

**Affiliations:** 1 Department of Civil Engineering, University of Burgos, 09001 Burgos, Spain; vrevilla@ubu.es (V.R.-C.); vortega@ubu.es (V.O.-L.); 2 Department of Construction, University of Burgos, 09001 Burgos, Spain; 3 Department of Chemistry, University of Burgos, 09001 Burgos, Spain; minago@ubu.es

**Keywords:** COVID-19 pandemic, lockdown, engineering course, higher education, online teaching, face-to-face teaching, student perception, university, autonomy, peer support

## Abstract

The intention of this study was to identify the elements that engineering students consider fundamental for successful learning on engineering courses. The aim was to provide generic guidelines suitable for any engineering course with which the teaching may be adapted in the light of comments from students, while student learning improves. The abrupt transition from face-to-face to asynchronous online teaching due to the COVID-19 pandemic prompted reflection among students on both teaching methods. Students were invited to evaluate each method through a survey of open-ended questions, identifying useful elements for their learning. The survey was repeated over nine weeks, to obtain the views of students after they had accepted the change and had critically analyzed how to improve online teaching. A cross-coded qualitative and mixed (word counting) analysis showed that the explanation of engineering concepts should be organized, hierarchical, repetitive, and exemplified. Furthermore, the teacher should link all the activities and projects to the concepts explained and quickly solve any doubts that they raised. As a consequence of the online teaching resulting from COVID-19, the need of independent student learning and peer support was also very evident. Teaching functions are essential on engineering courses, as teachers have to explain the overall concepts carefully, identify the key concepts, and demonstrate their industrial and professional applications. Furthermore, teaching methodologies that balance these aspects with autonomy and peer support for learning on engineering courses should be promoted.

## 1. Introduction

Universities currently offer a wide variety of engineering degrees. We find the traditional engineering degrees, such as architecture, and civil, agricultural, and industrial engineering, first taught at the cusp of the 20th century as the industrial revolution consolidated its hold on society. Mechanization moved on and the needs of society have also changed over the last century leading to the organization of engineering degrees that are advancing learning in new scientific areas such as Computer Engineering, Telecommunications Engineering, and Industrial Organization Engineering Degrees [1]. Such variable subject matter, from conventional to innovative sciences, has meant that engineering education is taught in many different ways and that no one model can ever guarantee that future engineers will learn and perform their future professional work to the best of their abilities [2]. Nevertheless, there are some aspects that are common to all engineering careers:

- Firstly, every engineering degree is multidisciplinary, i.e., the topics of the courses that students have to take are very varied [3]. Thus, in addition to courses on technical aspects, future engineers must study courses related to law, economics, and management. In addition, students are required to specialize in a specific branch of knowledge on many engineering degrees [4]. The clearest example is Industrial Electronic Engineering and Industrial Mechanical Engineering.
- Secondly, a key characteristic of engineering teaching is the need to balance the teaching of theoretical and practical concepts [5]. Although there are exceptions, it is difficult to find a completely theoretical course on engineering degrees, since the most common scenario is the subsequent application of theoretical aspects to examples of practical problems. Furthermore, any attempt to approach the practical examples with no initial explanation of the necessary theoretical considerations is generally impossible. All this means that a balance between theory and practice must be achieved to optimize course development and, therefore, student learning [6].
- Finally, engineering education is not usually confined to classroom activities. The ultimate goal of any type of engineering is to facilitate the needs of society, so that the concepts that students learn may be applied to the real world [7]. The teacher will often discuss examples of real cases and will organize field trips whose specific aspects are related to the course curriculum [8], thereby reinforcing the applicability and utility of the studies for students. A situation that also means that the profile of a teacher with work experience beyond the university is generally in great demand on engineering degrees, as they can offer students a vision that is closer to the work environment [9].

Traditionally, engineering teaching has been conducted through formal presentations where the teacher explains the theoretical concepts and then applies them to the resolution of a practical example [10]. The students do not participate too much when in the classroom, as they limit to take notes, and seek to understand the textbook explanations, the notes, and classroom explanations at home. This type of teaching methodology neither favors independent student learning, nor promotes their autonomy and motivation [5]. However, in recent years, innovative teaching methodologies have emerged, which galvanize student learning processes and have been shown to develop the three aspects listed above. Thus, for example, cooperative learning involves group work on practical cases, thereby balancing the theoretical explanations of the teachers with the shared understanding of students working in a group [11]. In addition, it also enables future engineers to develop group work skills, which are fundamental in professional practice [12]. Moreover, the teaching activities outside the classroom, visiting factories, companies, and civil works, among others, all facilitate the classroom presentations of the concepts and help students to approach the work environment where they will apply the concepts they have learnt [8]. Finally, multi-course experiences favor the student’s global learning process, as the links between apparently unrelated subject matter can often be clarified [13], thereby ensuring that the multidisciplinary approach of engineering will not paradoxically compartmentalize knowledge [14]. However, so many of these teaching methodologies never incorporate what students, in their own opinion, consider necessary to learn engineering. Sometimes, traditional teaching is even abandoned without clear insight into successful learning methods for engineering students. Therefore, students’ view on how to teach engineering appears fundamental.

The COVID-19 pandemic has provoked numerous changes within multiple areas of society, especially in the ways we relate to each other [15]. University education has also been affected by the need to maintain social distancing in the classroom, which has limited the size of student groups, increased online learning and distance tutorials, and the monitoring of activities outside the classroom [16,17,18]. However, the most intense impact associated with COVID-19 began with the pandemic. In a large number of countries around the world, strict lockdown was imposed, limiting mobility that even restricted access to the workplace, schools, and universities [19,20] with the aim of reducing the spread of the virus [21]. As a result, teleworking became popular and it was necessary to adapt the teaching of all university degrees that had traditionally been taught face-to-face, including engineering ones, to an online methodology [22,23].

Although the engineering students thought that learning during lockdown and teacher preparation for the situation was adequate [16], the adaptation to online teaching implied an abrupt change in the way students were used to learning [24]. It was due to the specific features of online teaching, such as the absence of direct contact between teacher and student, imbalanced progress between theoretical and practical exercises, the need for greater responsibility from the students with regard to their own learning, and the great dependence on the quality of the teaching material for proper understanding of the concepts among students [25]. Having been accustomed to face-to-face teaching, the contrast of a sudden change to online teaching undoubtedly meant that students gained an interesting perspective on engineering teaching, and identified the strengths and weaknesses of both traditional face-to-face teaching and online teaching during the lockdown.

According to the above, this study aimed to identify the aspects that students consider fundamental for learning in engineering careers. To that end, advantage was taken of the lockdown imposed following the outbreak of the COVID-19 pandemic, during which face-to-face university courses were taught online. After selecting a representative sample of engineering degrees and courses of the University of Burgos, Spain, students were asked to complete the same survey, consisting of five open questions, during each of the nine weeks of online teaching. The structure of the survey and the novel and unexpected online teaching methodology to which they were becoming accustomed over time encouraged a reflective and critical spirit among the students that helped them to recognize the fundamental aspects for the successful learning of engineering. Thus, the ultimate goal of this study was to provide any engineering teacher with simple and clear guidelines on how to orient their teaching, in order to achieve successful learning based on the viewpoints of the students.

## 2. Materials and Methods

This section describes the design of the experiment and the points that are necessary in order to understand the results properly.

### 2.1. Description of the Study

The COVID-19 outbreak, and the fast spread of the virus in the city of Burgos, Spain, led the University of Burgos to suspend face-to-face teaching on Thursday, 12 March 2020 at 3:00 p.m. On Saturday, 14 March 2020, a State of Emergency was declared in Spain, according to which a strict confinement of the population was established to control the transmission of the virus. This confinement led to the closure of all schools and universities in the country, so that the rest of the 2019/2020 academic year was taught online. The Rectorate of the University of Burgos urged teachers to continue teaching as normally as possible and teaching was set to resume online on Monday, 16 March 2020.

The four days of adaptation to online teaching available to the teachers, from 12 to 16 March 2020, were key, as the general guidelines for the development of this study were defined. During this period, the authors of this study conducted the following activities:

- The conceptualization of the study and the clear definition of the aspects of engineering teaching that were to be evaluated.
- The preparation of the survey to be administered to the students.
- The selection of the courses participating in this study and the definition of the study sample.
- The establishment of general guidelines common to all participating courses on how to teach online. In this way, all courses would be taught in a similar way and the non-controlled variables introduced in the study could be minimized (Fulton 2020).

#### 2.1.1. Objectives and Scope of the Study

Although many aspects of teaching might have been studied during the exceptional situation of confinement, the authors decided that a general approach was best, seeking results of utility within the broad field of engineering teaching. They therefore decided to invite students to define the essential aspects of an engineering course conducive to proper learning. The question was not directly framed, as it might otherwise have been answered with stock or standard answers, without deep and thoughtful reflection [26]. Instead, it was decided to take advantage of the situation provided by the confinement in two ways:

- First, the sudden change to online teaching had taken students outside of face-to-face teaching, outside of their “comfort zone”, and they had to accustom themselves to a very different teaching methodology in many ways compared to face-to-face teaching, such as contact with the teacher, independent study, and the way both theoretical and practical concepts were explained [27]. This induced in students initial reflections on the beneficial elements of online teaching and what they missed from the face-to-face sessions. A survey was therefore designed to stimulate student reflections on conventional face-to-face teaching and then to compare it with the online teaching during lockdown. Finally, following their reflections, the students were asked what they needed to learn engineering successfully.
- Second, it was decided to use “time” as a main variable. Faced with undesirable changes to their lives, people will often react adversely and rebel. Subsequently, they will typically express resignation, before fully accepting the change. Finally, they may begin to evaluate the new situation and to engage in a critical analysis of how the situation could be improved [28]. It was therefore decided to administer the survey to students once a week during the online teaching period. The intention was to detect evidence of those three phases—rebellion, resignation and acceptance, and adaption and reflection—and to analyze the responses from the students once they had reached the reflective stage. In this way, the students completed the survey each of the 9 weeks that the online teaching lasted (confinement period). The students filled out the survey at the end of the week (on Friday) so that, when responding, the students had the complete picture of the teaching activity carried out during the week in the course.

Having defined the objective of the study, the aspects to be studied and how relevant results may be obtained, the survey was prepared.

#### 2.1.2. Instrument: Survey

The survey was designed by the authors of the study following the guidelines of other similar studies [4,29] and with the support of colleagues who are experts in the development of this type of surveys.

The survey consisted of five open questions that students answered throughout each of the nine weeks of online teaching. These questions, with no word limits, were mainly intended for all students to answer, highlighting all aspects considered relevant. The development of a closed-ended survey could lead to a restriction and limitation of the aspects underlined by the students to those considered relevant by the authors of the study [5]. However, although most of the answers requested from the students were open-ended, some of them were numerical or bi-optional questions, so they required quantitative or bi-optional responses. Their primary purpose was to detect the three phases of behavior when facing a sudden or undesirable change indicated above [28].

The survey was designed in Spanish and, likewise, the students’ responses were conducted in Spanish. However, for an optimum understanding, both the survey and the results are translated into English throughout the article. The five survey questions were:

1. Rate your learning on the course this week from 1 to 10. Briefly justify your response.
2. From a personal point of view, do you think you have learned more during this time of online teaching or when you had face-to-face classes?
3. Linked to the previous question, do you consider that the adaptation of the course to the online mode has been successfully performed?
4. What aspects would you improve or change regarding the online teaching you are receiving? Do you consider that online teaching has any advantage over face-to-face teaching?
5. You have experienced both face-to-face and online teaching. What do you need from each teaching methodology to learn on any engineering course? Do you believe that both the online and the face-to-face teaching you have received has facilitated your learning process? Briefly justify your response.

**Figure 1:**
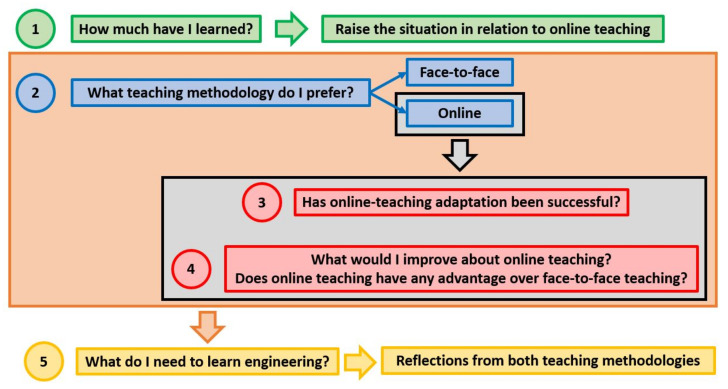
Schema of thought processes when completing the survey.

The first question of this survey was intended to ask students to consider how they had learned during that week, raising the situation of online teaching that they were experiencing. Subsequently, questions 2, 3, and 4 were intended to stimulate student reflection on the advantages and disadvantages of each type of teaching regarding their learning, comparing both teaching methodologies. In question 5, the students were asked to indicate what might be needed for quality teaching, so that they could successfully learn based on their experiences during both face-to-face and online teaching. Questions 1 and 2 were mainly used to identify the three phases of behavior explained in Section 2.1.1. The schematic thought process followed to complete the survey is shown in Figure 1.

Participation in this survey was voluntary and the data obtained were anonymously processed, aspects that were communicated to the students of the participating courses before the start of the study.

#### 2.1.3. Role of the Teacher

The courses involved in this research were subsequently defined. To that end, the researchers in charge of this study randomly contacted numerous teacher staff associated with the engineering degrees taught at the University of Burgos. The objective was to recruit as many participants as possible in a random way, so that the results might have a broad scope of application and ensure that the study approached the study of engineering from a general approach. When contacting the teachers, the objectives of the study and their roles were briefly explained:

- Teachers had to be imparting online teaching according to the guidelines established for the study. In this way, it was possible to minimize non-controlled variables that might affect the results [27].
- At the beginning of the online teaching period, they had to explain the study and its objectives and the conditions for participation (completion of the survey, timeframe, etc.) to the students. In addition, the students had to be informed that any participation would be completely voluntary, with no consequence for their grade on the course, and that all their responses would be treated anonymously.
- Remind students that they could answer to the survey weekly. This action was intended to muster as much participation as possible from the students.

Overall, 6 teachers agreed to participate in the research, who carried out the study in 5 different courses. The disparity between the number of teachers and courses was because there was a course taught by two teachers. The level of participation was considered adequate given the exceptional conditions caused by the COVID-19 pandemic, the corresponding confinement and the consequent need for teachers to balance family and professional life [30]. The five participating courses covered different Bachelor’s Degrees, such as the Bachelor’s Degree in Agroalimentary Engineering and the Rural Environment and Bachelor’s Degree in Civil Engineering, and Master’s Degrees, such as the Master’s Degree in Civil Engineering.

The demographic characteristics of all participants, both teachers and students, are detailed in Section 2.2.

#### 2.1.4. Guidelines for Online Teaching

Some general guidelines for online teaching were defined for the teachers of the participating courses, to minimize the appearance of unexpected variables in the study. During the process of contacting the teachers, as described in the previous section, they were all asked to affirm that they had the necessary knowledge and skills to provide online teaching in keeping with the characteristics defined for the study. To that end, they were provided with all the necessary support. The guidelines established for online teaching were as follows:

- The online teaching had to be asynchronous, which meant that, in addition to the provision of the corresponding PDF files and presentations, it was taught through videos in which both theoretical and practical concepts were explained [31]. Students would not be required to be present at a specific time for online classes through tools such as Microsoft Teams, Skype, or Zoom. This way of teaching facilitated the balance between work and family–social life both for the teachers, who would have a completely free schedule to prepare the necessary videos, and for the students [32]. In addition, asynchronous online teaching would mean a greater change for students compared to face-to-face teaching, which could imply addressing a wider range of aspects in their answers to the survey [33], as well as deeper reflection on engineering teaching.
- Communication between teachers and students had to be continuous. This rule was intended to give students the feeling that the teachers cared about their learning, an aspect that has always facilitated learning in online teaching [34]. Thus, teachers were asked to continuously inform students about new teaching material uploaded to the teaching support platform UBUVirtual (Moodle) and to answer students’ doubts posed by email or through that platform as quickly as possible. Additionally, they were asked, depending on their availability, to set a schedule for online tutoring (Microsoft Teams, Skype, Zoom...) with students once a week. Although not mandatory for students to connect to those tutorials, it provided a forum for students to ask direct questions to the teacher.

No course grading guidelines were established, since the aspects included in the course Teaching Guide had to be respected, as indicated in the Teaching Regulations of the University of Burgos [35].

### 2.2. Participants

The 6 participating teachers (5 courses), 4 men and 2 women, had a mean age of 43.82 ± 11.39 years. All of them had completed a Master’s degree in engineering.

All the students of the 5 participating courses were asked to take part in the study by filling out the survey. Those students who participated did so voluntarily. The sample consisted of a total of 66 students, with a mean age of 21.59 ± 2.47 years. No distinction was made between sex, age or course. The objective was to obtain results of a general character with a broad scope of application.

### 2.3. Analysis of Results

The responses were analyzed using various methodologies to ensure that all aspects highlighted by the students were adequately detected:

- First, a quantitative analysis was used to analyze the answers to the first part of the question 1 of the survey. This analysis consisted of obtaining the confidence intervals of the level of learning for each week of the study.
- Second, the answers to questions 2 and 3 were analyzed as qualitative statistical variables by obtaining frequencies. The three phases of behavior described above (Section 2.1.1) could be clearly detected in the results of questions 1 and 2, apart from providing relevant information on student learning [28].
- Most of the answers of the students (second part of question 1, and questions 4 and 5) were qualitative, so the research team analyzed them using a methodology of cross-coding and continuous feedback. Hierarchizing, grouping, and generalizing the aspects that the students addressed were possible with this approach, so that general conclusions could be drawn from particular opinions [36]. The ATLAS.ti software(Scientific Software Development GmbH, Berlin, Germany) was used.
- Finally, for question 4, a mixed analysis based on word counting was also performed using ATLAS.ti. The evolution of the number of times that a certain term was cited gave some insight into its relevance among the students over the various weeks of the study [37].

In this analysis, no distinction was made between students of different courses or by gender or age. The objective was to obtain conclusions that were as general as possible [16]. All these forms of result analysis are discussed in the Results section.

## 3. Results

The results from the analysis of the answers from the students are presented in this section. In addition, an analysis and some discussion of the results are also provided.

### 3.1. Level of Participation

**Figure 2:**
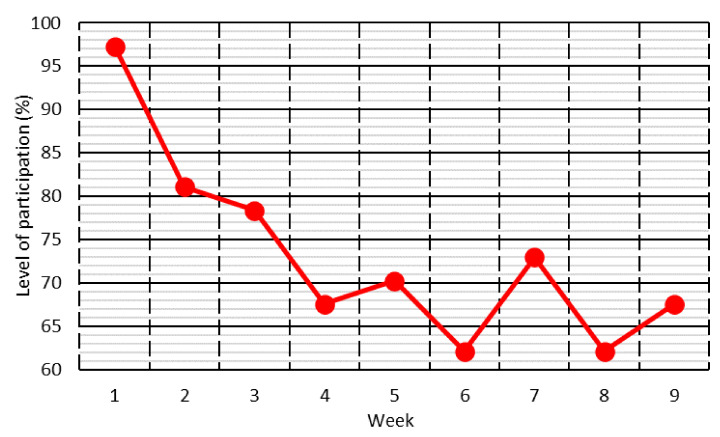
Level of participation throughout the study.

Figure 2 shows the level of student participation throughout the nine weeks of the study. Initially, participation was very high, but subsequently decreased to 60–75% after the Week 4 of online teaching. This behavior corresponded to the usual tendency of an ongoing study that depends on the participation of a certain population group. Initially, the study arouses attention and the level of participation is high. Later on, some people lose interest in the study and drop out, so that only those who considered it useful and relevant continued to participate [38].

The levels of participation achieved meant that the survey was answered weekly by 40 to 50 students. This sample size was considered adequate for the purpose of the study, considering the difficult conditions, especially psychological, caused by the strict confinement of the population [15].

### 3.2. Learning Assessment: Question 1

**Figure 3:**
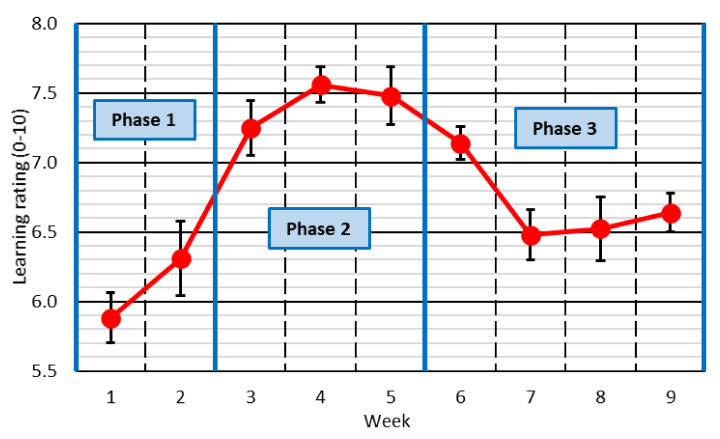
Learning rating throughout the study.

The first question of the survey asked students to rate their learning on a scale from 0 to 10 throughout the corresponding week. The mean values and the confidence intervals of the students’ answers are shown in Figure 3.

The worst ratings of the learning level were obtained in the first two weeks of the study (rating around 6 out of 10), and then suddenly increased, reaching a learning rating of around 7.4–7.5 from Week 3 to Week 5. Subsequently, the learning rating decreased and was around 6.5 out of 10 during the remaining weeks of the study. This behavior in the assessment showed the three above-mentioned phases of behavior through which a person generally goes when facing a sudden change [28]:

- Phase 1: Rebellion. The person cannot accept the change. This refusal led to the worst learning ratings in the first two weeks.
- Phase 2: Resignation and acceptance. In this study, this phase was associated with students considering that they had reached a high level of learning between Weeks 3 and 5.
- Phase 3: Adaptation and reflection. Once the person adapts to the change, they critically evaluate the situation, reflect on it, and try to improve it. This phase is identified by a noticeable decrease in the level of learning from Week 6–7 onwards.

These three phases of behavior were also reflected in the students’ comments to justify the assessment of their learning level, as extracted from the qualitative analysis that was performed.

- During the first phase of behavior (Weeks 1 and 2), students clearly showed their dissatisfaction with the existing situation and the way in which online teaching was being conducted.

*“I am not convinced by the way the course is taught [...], the class should be taught in real time” “[...] I cannot make summaries while I listen to the teacher, which is how I have always studied” “The way of adapting to online teaching has been very bad [...] there is no clear learning rhythm as in the face-to-face teaching” “[...] I am having a hard time concentrating, I have to establish my own rhythm and it is not easy for me” “[...] all the teachers are determined to send a lot of work, because there is no class [...] I am overwhelmed”* (Weeks 1 and 2, rebellious phase).

- From Week 3 to Week 5 (second phase of behavior), there was a predominance of responses in which the students showed a great conformity. These opinions basically consisted of accepting the online-teaching methodology completely, without finding any problem.

*“[...] the videos that the teachers are making are well explained [...] and make up for the lack of face-to-face teaching” “[...] relevant teaching material is continuously being uploaded, well done and important for the course” “The material uploaded and the availability of the teacher is very good [...] I understand all the concepts correctly [...]” “[...] the theoretical part of the course is understood very well, because the most learning can be done by ourselves looking for information [...]” “[...] once you get used to it you see that you can also learn with this way of teaching”* (Weeks 3, 4 and 5, acceptance phase).

- During the last four weeks of the study, the students showed a more reflective attitude, so that the decrease in the consideration of the learning level was due to the identification of aspects that could be improved. It was observed that the students offered no solution in their responses to the aspects that had been criticized, which justified the need for question 4.

*“[...] more continuous contact with the teacher is necessary [...] only one day a week is not enough” “[...] the experience of the teachers is indispensable [...] this way I have to do much more research on my own and although it is useful it is more difficult to understand everything well” “[...] you can study the theoretical part on your own [...] but you need the practical part to be explained to you [...] if conditions change the problem can be completely different and asking to the teacher is key [...]” “[...] the workload should be controlled more precisely [...] online teaching does not justify that the workload is greater than when teaching is face-to-face [...]”* (Weeks 6, 7, 8 and 9, reflexive phase).

The three behavioral phases detected (rebellion; resignation and acceptance; adaptation and reflection) should be considered when implementing any modification of any teaching methodology. This adaptation should seek to minimize rejection of the new teaching methodology during the rebellion phase, as well as student conformism during the phase of resignation and acceptance. A simple solution that may prove to be successful is to adapt the content of the course covered in each phase and promote the students’ participation:

- During the rebellion phase, it may be interesting to reduce the number of concepts explained. This will allow emphasizing more on the concepts addressed. In this way, even if the students show a less proactive and more reticent attitude towards learning, the greater repetition and time dedicated to each concept can guarantee that the quality of their learning is not affected. In addition, small changes could be introduced to increase student participation (collaborative learning, group work...), since it has been demonstrated that students perceive greater learning when they are allowed to participate actively [5,39].
- During the phase of conformism and acceptance, the concepts not explained during the phase of rebellion to dedicate more time to each concept could be addressed. Furthermore, it would be advisable to promote at all times a reflective and critical attitude on the part of the students about the concepts explained. In the conformism phase, the student accepts the teaching methodology, without seeking to improve it. Reflection on the concepts explained in class can encourage students to reflect on how what has been explained can be learned more satisfactorily, as shown in other studies of the bibliography [26,40].

### 3.3. Teaching Methodology with Higher Learning Level: Question 2

**Figure 4:**
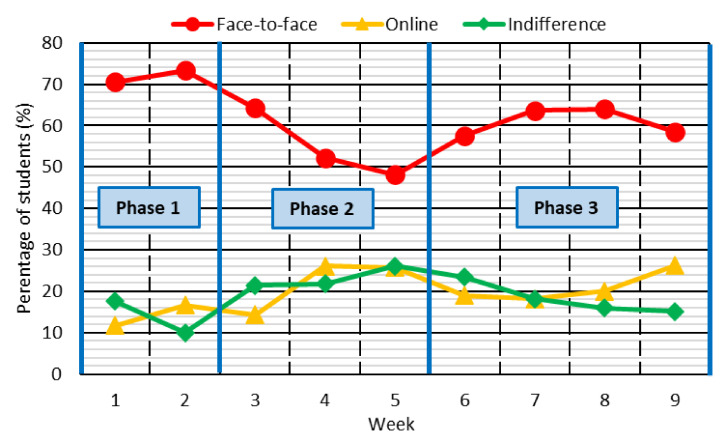
Preferred teaching methodology.

The temporal duration of the three behavioral phases described in the previous section was corroborated through question 2 of the survey. In that question, students had to indicate their preferred teaching methodology: online, face-to-face or indifference. Figure 4 shows the percentage of students who preferred each teaching methodology.

Throughout the study, students showed a clear preference for face-to-face teaching, which was also the teaching methodology they had always experienced. No great difference was detected between the percentage of students who preferred online teaching or who were indifferent towards the type of teaching they had received. It was only in the last two weeks of the experiment that a greater number of students were observed to be in favor of online teaching, possibly because, albeit slowly, they were getting used to receiving that type of teaching or because the online teaching was more appropriate.

Regarding the duration of each phase of behavior, the results in Figure 4 were in accordance with the duration of each phase established in the previous section. At the beginning the students showed a strong preference for the face-to-face teaching, but after the Week 3, they began to think that face-to-face teaching was not so advantageous. In fact, in Week 5, fewer than half of the students considered face-to-face teaching to be better and indicated that they preferred online teaching or that they were indifferent to the teaching methodology. Finally, from Week 6 onwards, there was an increased preference for face-to-face classes in a more sustained manner, showing the reflective-behavior phase.

### 3.4. Adaptation of the Course to Online Teaching: Question 3

This question was linked to the previous one, which was intended, once the preferred type of teaching had been indicated, to encourage reflection on the online teaching.

**Figure 5:**
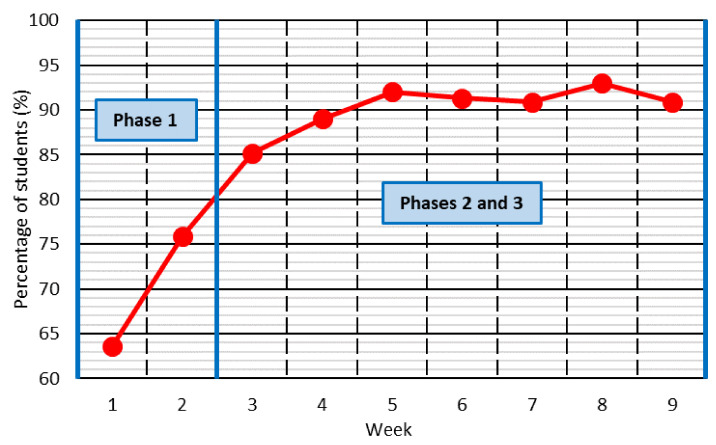
Percentage of students who considered successful adaptation to online teaching.

Figure 5 shows the percentage of students who considered, for each week of the study, that the adaptation to online teaching had been adequately implemented. That question was unlike question 2 (Figure 4), which led to notable differences in the results, as some students preferred the face-to-face classes, but at the same time considered that the online teaching had been adequately implemented. It can be observed that students initially indicated that the adaptation was not successful (rebellious phase). However, the percentage of students who considered that the adaptation had been adequately implemented increased over time, stabilized at around 90% from the Week 5 onwards. Two conclusions can be drawn from this trend:

- First, there was no difference between the behavioral phase of acceptance (resignation) and the reflective phase regarding this issue. Therefore, although students began to be more critical about the online teaching from Week 6 onwards, because they had started to reflect upon the way to improve this type of teaching, they considered that, in view of the exceptional situation caused by the COVID-19 pandemic [19], the adaptation had been adequately implemented. Students showed great maturity when analyzing this aspect.
- On the other hand, both types of teaching offered useful features for successful learning in engineering. Thus, although most students preferred face-to-face teaching (Figure 4), online teaching was also beneficial in some respects.

### 3.5. Weaknesses of Online Teaching and Its Comparison with Face-to-Face Teaching: Question 4

The percentage of students who considered the adaptation to online teaching to have been successful remained approximately constant after Week 5 (Figure 5), an aspect that meant that the vast majority of them found positive aspects in online teaching compared to face-to-face teaching. However, it should be noted that the preferred type of teaching was generally the face-to-face methodology (Figure 4), thus students also considered that many elements of face-to-face teaching were fundamental for engineering learning.

**Figure 6:**
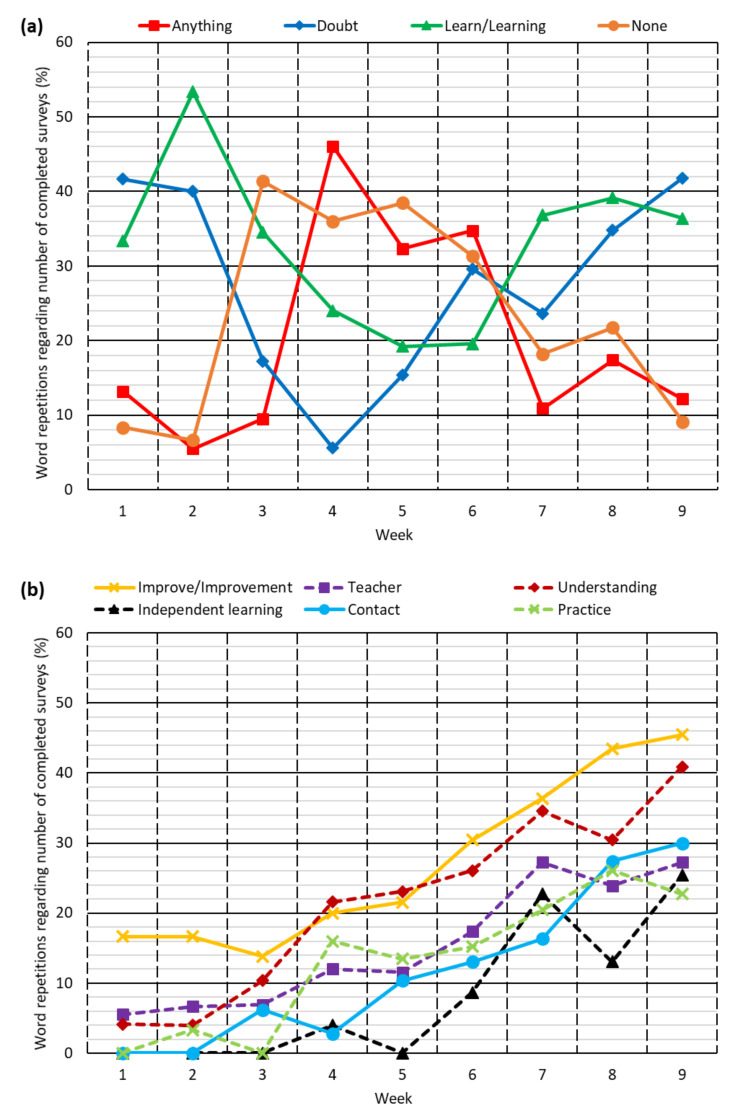
Word counting for answers to question 4: (**a**) words linked to the three phases of behavior; (**b**) words showing the appearance of reflective attitudes among students.

First, a mixed analysis was performed based on word counting [37] of all responses from the students. The repetitiveness (times the word was cited in relation to the number of students who completed the survey each week) of the ten most cited words by the students that were considered most relevant is shown in Figure 6. Two different trends can be distinguished for these words:

- On the one hand, four words (*anything*, *doubt*, *learn/learning* and *none*) reflected the three phases of behavior described [28]. *Anything* and *none* were hardly mentioned in the first weeks due to the rebelliousness of the students towards online teaching, underlining that, in the students’ opinions, everything needed to be improved. Subsequently, those words became the most frequently used in the second phase of behavior, in which students showed acceptance. At the beginning of the reflective phase, their use decreased again. The words *doubt* and *learn/learning* showed exactly the opposite trend, highly cited at the beginning and then their citation decreased and increased again. The use of these words also shows that learning and the resolution of doubts were complaints during the rebellious phase, which became elements upon which the students later reflected. It all shows that, although the answers to question 3 never reflected the three phases of behavior, those phases, detected in questions 1 and 2, also appeared when the students compared face-to-face and online teaching.
- The use of the words *improve/improvement*, *teacher,* and *understanding* increased as the weeks passed by, especially after Week 6, due to the emergence of reflective attitudes among the students. The words *independent learning*, *contact,* and *practice* showed the same trend, with the difference that their use in the first weeks was practically nil.

In view of the results obtained through the mixed analysis, it was decided not to consider the responses to question 4 of the survey from Weeks 1 to 5 (behavioral phases of rebellion and acceptance), so that an exhaustive qualitative analysis of the responses was carried out during the reflective phase (Weeks 6, 7, 8, and 9). During the first two behavioral phases, the predominant number of responses was clearly against online teaching, some of which are shown below:

*“It is essential to have live classes [...] we are making a living.” “We must try to have an online class as similar as possible to a face-to-face class [...]” “[...] it cannot be that what I learn depends exclusively on me [...] the teacher’s role must be more relevant [...]” “[...] I need to ask my doubts to learn the concepts [...] email is not enough.”* (Weeks 1 and 2, rebellious phase).

*“At the moment I would not change anything, [...] we can monitor the course with the available material on the platform and ask any questions that may arise by emailing the teacher.” “At the moment, I cannot think of any aspect that could be improved” “I think the teaching is quite effective [...] it’s the best way to teach online.” “I don’t know which aspects I would improve [...] and I don’t know which options there might be for improvement [...].”* (Weeks 3, 4 and 5, acceptance and resignation phase).

The qualitative analysis conducted on the answers to question 4 during Weeks 6–9 (reflective phase) comprised a total of 379 text extracts, which were cross-coded. In addition, there was continuous feedback from the authors of the study during the analysis, so that all relevant aspects were identified and prioritized. An analysis in which all the shortcomings and benefits of online teaching compared to face-to-face teaching [14] surfaced, posing a clearly reflective state for students to address question 5 of the survey.

Regarding the aspects of online teaching (described in Section 2.1.4) that they would improve, the students basically highlighted three aspects:

- On the one hand, the students indicated that online teaching requires a larger number of exercises to be independently solved. They indicated that, as there was no direct contact with the teacher, the explanation was not so clear and they needed more exercises to reinforce the practical concepts, even if the exercises were not explained and only the wording and the solutions were provided.

*“I think there should be some more practical exercises to practice independently on a voluntary basis [...] in this way we would reinforce the concepts.” (Week 6). “[...] I would increase the number of exercises that we are asked solve [...].” (Week 8). “[...] I’d like to promote independent learning [...] in the practical part I would like to have more exercises to solve.”* (Week 9).

- The students also highlighted that online teaching required very detailed organization, in so far as each material had to be uploaded in the right order and the completion dates of the proposed projects had to be fixed, for effective course follow-up. In addition, continuous notifications from the teacher of the material uploaded onto the platform were convenient, so that students were aware of all the available material. The material supplied for online teaching was much more abundant than in the face-to-face class, which also fostered the same need. In general, the students indicated that the lack of continuous communication with the teacher meant that the follow-up of the course was not so efficient.

**Good organization of the course:***“Proper explanations of how to carry out the practical assignments that must be delivered are necessary and the explanations with the online teaching were not as good as with the face-to-face classes [...].”* (Week 7). *“[...] it would be convenient to establish from the beginning all the delivery dates for the assignments, so that we could better organize ourselves.”* (Week 9). *“[...] the follow-up of the exercises should coincide with what is explained in theory [...] in the face-to-face classes, it is easy to situate yourself, even if you have seen the theory corresponding to the exercises some time ago.”* (Week 8).

**Teaching material:***“There is so much teaching material [...] the teacher should explain the contents of each folder as soon as it’s uploaded [...].” (Week 9). “[...] providing the exercises right after explaining the theory would make it much easier to understand [...].”* (Week 6).

- Finally, students indicated that it was necessary to increase contact with teachers, the main difference between online and face-to-face teaching. This contact was necessary to resolve complex doubts and highlight the most important theoretical aspects, especially useful for the resolution of exercises. Unexpectedly, students also highlighted the need for continuous contact with their peers, indicating that doubts are often solved with the support of classmates.

**Contact with teachers:***“[...] I miss the teacher’s real examples [...].”* (Week 6). *“The teacher’s experience of how the concepts presented in class are really applied is fundamental [...] that’s been lost with online teaching.”* (Week 8). *“[...] I would like more time to ask to the teacher more questions, especially about the practices.”* (Week 9). *“[...] highlighting the most important theoretical aspects is very useful [...] it not only helps to study for the exam, but also to solve the problems [...].”* (Week 8). *“[...] I would like the teacher to clearly indicate which theoretical aspects are the basis for solving the problems [...].”* (Week 7).

**Contact with peers:***“[...] meetings with classmates should be encouraged [...] doubts are often solved this way [...].”* (Week 6). *“[...] I cannot ask my classmates simple doubts that I don’t dare ask the teacher [...].”* (Week 9).

Although some students found aspects to improve in the online teaching that they were receiving, they also recognized that some characteristics of that teaching methodology were beneficial to their learning. These aspects were:

- Firstly, many students indicated that online teaching allowed them to solve some doubts more quickly. Although they considered the contact to be insufficient for resolving complex doubts (an aspect explained above), the students were grateful that simple doubts were resolved very quickly, mainly by email. They agreed that they had to wait longer for simple doubts to be cleared up in face-to-face teaching.

*“[...] the connection with the teachers to solve simple doubts is the right one [...].”* (Week 6). *“I know that it is because we are all locked up at home but I love the speed with which the doubts are solved by email [...] although for more difficult doubts mainly related to the practical exercises, face-to-face class is better.”* (Week 8). *“[...] the teacher is available all the time by email and solves the doubts very quickly.”* (Week 9). *“[...] I asked a question by email and received an answer within two hours [...].”* (Week 9).

- Secondly, the students also appreciated the flexibility of the teaching they had received. On the one hand, they were referring to the delivery of projects and questionnaires, in which the teachers, without any indication from those in charge of the study, generally assigned time periods for their delivery, an unusual practice in face-to-face teaching in which a specific date is fixed [34]. On the other hand, this flexibility also referred to the explanation of both the theoretical and practical concepts, since the use of videos allowed the students to watch the explanation as many times as they wished until they had understood them all. Although students continued to have doubts, they indicated that they could make a greater effort to understand the contents of the course on their own.

**Projects delivery:***“One of the main advantages is to have a time interval to hand in the projects [...] I think that this has removed the rush to deliver.”* (Week 6). *“[...] the delivery of the projects has been flexible [...] it has allowed me to go deeper into the topic of the project, you can do it better and learn more.”* (Week 6).

**Use of videos:***“Videos are a fantastic teaching material [...] they can be rewound in case you don’t understand something.”* (Week 8). *“[...] it is very advantageous to be able to watch the videos as many times as you may need, until you understand everything [...] in face-to-face teaching I can’t make such an effort to understand the concepts on my own and I end up asking a lot of questions.”* (Week 9).

- The last aspect highlighted by students was independent learning. Online teaching implies that learning depends to a greater extent on the work of the students, since their contact with the teacher is no longer continuous within a classroom [27]. Furthermore, this independent learning was promoted by the use of asynchronous online teaching [32], without real-time classes, and resulted in the student making greater effort to search for information not only to complete the projects, but also to understand the concepts.

*“What you learn in online teaching depends more on the student’s work [...].”* (Week 6). *“[...] if you make an independent effort, the learning can be at the same level or higher than that achieved face-to-face [...].”* (Week 7). *“[…] we are learning on our own, we don’t depend a lot on anyone […] I think I understand things better this way.”* (Week 8). *“[...] I had to look for additional information to work on class topics and projects [...] and I was able to understand many of the concepts by myself.”* (Week 8). *“Researching different sources to understand everything that had been explained meant that I learned a lot [...] it is something that I would never have done in face-to-face teaching.”* (Week 9).

### 3.6. Tips for Successful Learning in Engineering Courses: Question 5

After reflecting on the advantages and disadvantages of online and face-to-face teaching, the survey ended by asking students what they needed to learn engineering successfully. Based on the aspects indicated above, only the answers provided by the students during the reflective phase, from Week 6 to 9, were considered relevant. The answers were qualitatively analyzed by cross-coding, considering 7 main codes that represented the 7 aspects that the students considered relevant for adequate learning. This coding was completed with continuous feedback from the authors of the study. A total of 424 text fragments were analyzed.

The first aspect that the students emphasized was that the course should be correctly explained, organized and hierarchized in order to achieve optimal learning, which is usually fundamental in all types of courses, not only in engineering [22]. This comment referred to the fact that both theoretical and practical documentation should be correctly presented and explained. In addition, both aspects should be addressed simultaneously, i.e., practical exercises immediately after the explanation of the necessary theoretical concepts [41]. This organization should also be reflected in the proper organization of the proposed projects. Finally, they also indicated that the most important concepts should be highlighted throughout the course, as this allows the construction of the course building, in which these key aspects would be the pillars.

**Appropriate explanation of concepts:***“[...] the teacher should explain everything in detail and highlight the key aspects of the course [...].”* (Week 7). *“The teacher’s interest is fundamental [...] you need complete notes and a detailed explanation.”* (Week 9). *“The teacher must highlight what is most important [...] it allows you to know where to start studying.”* (Week 9). *“Carefully prepared documentation makes learning much easier [...].”* (Week 6).

**Simultaneous explanation of theory and practice:***“[...] both theory and practice should be explained in a simple but complete way [...].”* (Week 6). *“Theory and practice must be approached in a coordinated way [...] you cannot set a problem on something when the theory was explained a month ago [...].”* (Week 8). *“[...] I would recommend doing the exercises of a course as soon as you explain the corresponding theory [...].”* (Week 9).

**Projects delivery:***“[...] the assignments that the teacher asks for cannot be done on the fly [...] they must be planned in advance [...].”* (Week 7). *“[...] knowing the projects you have to do from the beginning makes things much easier [...].”* (Week 6).

Secondly, it was noted that students referred to another aspect that is also usually required in all types of courses and which is closely linked to the previous aspect: the course should be adapted to the concepts that are explained. This adaptation is related to two aspects. On the one hand, all teaching material should be adapted to the nature of the course, so that, if the course has a high practical content, as in many engineering courses, the notes should be in line with this, containing precise explanations of the aspects for practical application, not only of the theoretical concepts [16]. On the other hand, all the requested assignments should be related to the concepts addressed in class and should go deeper into them. The assignments that were sometimes requested on complementary topics were never considered to have contributed much to the learning of the course.

**Teaching material preparation:***“[...] notes that address both theory and practice are the best [...] sometimes the notes explain only the theory and the exercises are explained viva voce [...].”* (Week 8). *“[...] the notes should explain both theory and problems [...]”.* (Week 9).

**Projects linked to concepts addressed in class:***“The teacher must stick to what is explained in theory when approaching the exercises [...].”* (Week 6). *“[...] we need projects that address what we have been taught in class [...].”* (Week 6) *“[...] everything we do should be related to what we learn in class [...] requesting extensive projects on unexplained topics gives the impression that the teacher doesn’t know how to teach the course [...].”* (Week 7). *“[...] we need projects that apply what we have studied in class [...] projects that are unrelated to the classroom explanations don’t contribute much to the course.”* (Week 8).

The third element that students considered necessary on any engineering course was the teacher’s support. Teachers should not limit their role to simple explanations of theoretical concepts or exercises, but should also be active in student learning, ensuring that, once explained, the concepts have been properly understood. In addition, the teachers should be available to solve any doubt that students may have. The students mentioned that, although teachers may assume that something is known from previous years during a class, they should be available to explain it, if questioned on the matter in tutorials, for example. Engineering courses are often characterized by their high practical content and the close link between theory and practice [5]. Therefore, a correct understanding and learning of practical concepts implies that any doubt about them, however simple it may seem, should be solved [4].

**Teacher’s attitude:***“[...] teachers should get involved, check that we understand what they explained and help resolve any doubts we have [...].”* (Week 6). *“Teachers should monitor whether students are understanding what has been explained [...].”* (Week 6).

**Solving doubts:***“[...] I understand that the teacher takes certain things for granted [...] but when you ask them, they should be willing to explain it to you, even if it is after class or in tutorials.”* (Week 7). *“[...] it is more important that what the teacher explains is well understood, resolving our doubts, than explaining the whole syllabus [...].”* (Week 8). *“[...] teachers should be available outside the classroom to resolve all doubts [...].”* (Week 8). *“[...] doubts by email should be resolved quickly, as has happened during online teaching.”* (Week 9). *“[...] teachers should resolve any doubts we have and care about whether we understand everything [...].”* (Week 9).

The teacher’s role is also fundamental in the fourth aspect addressed by students: the course concepts that are taught should have proper examples and their scope of application should be clearly shown. The purpose of any type of engineering is to respond to the needs of society [38]. Therefore, the concepts that engineers learn during their training will certainly be applied in a practical way during their future professional work [9]. Thus, students indicated that it would be convenient if all the concepts explained in class were related to real examples, as students could then understand the applications of what they were studying in a simple way and how the concepts that they are studying will be useful during their professional work.

*“Teachers know the professional world [...] they should show us how what we are studying is applied in the professional world.”* (Week 6). *“It is not only necessary to explain the theory [...] putting realistic practical cases is very useful to understand what is explained [...] they also show you how you will use those concepts in the future.”* (Week 7). *“[...] it is necessary to explain the concepts in such a way that they are fully understood, for example, through images of real cases.”* (Week 8). *“[...] it would be useful for teachers to show us how we will use what we study [...] with a formula we calculate something, but what do we do with what we have calculated?”* (Week 8). *“[...] every time teachers give a practical example from the professional world, I pay much more attention [...] I am interested in finding out the real applications of what I am studying [...].”* (Week 9).

Fifth, the students considered that the repetition of concepts would favor their learning in view of the asynchronous methodology used in online teaching. Therefore, any engineering course should be based on repeatability. Students indicated that the repetition in different classes of the key concepts that were explained meant that those concepts could be periodically remembered, so that they were better learnt, a strategy that could be similar to the possibility that students had of repeating the explanatory videos provided during the online teaching. This aspect is closely linked to the criterion of organization and prioritization mentioned above, once again highlighting the importance of identifying and conveniently explaining to the students the key concepts of the course [14]. They also indicated that briefly recalling at the beginning of any class what was explained in the previous class gave a better understanding of the new concepts that had been explained.

*“All the topics have to be clearly explained [...] the teacher has to insist on the most important aspects throughout the course [...].”* (Week 6). *“[...] what is important should be repeated several times [...].”* (Week 7) *“[...] I don’t like it when the teacher doesn’t go over the essential aspects several times [...].”* (Week 8). *“[...] I could watch the videos during online teaching repeatedly until I understood all the concepts [...] something similar in face-to-face teaching would be very useful [...].”* (Week 8). *“[...] repeating the most important concepts from the previous class at the beginning of each class helps me to situate myself, so that I can learn better the new concepts that will be explained [...].”* (Week 9). *“[...] the teacher’s linking of the concepts to be explained in class with those of previous classes greatly facilitates the understanding of the course [...].”* (Week 9).

The penultimate relevant aspect mentioned by students, independent learning, was outside the usual practice of conventional teaching. Thanks to online teaching, students experienced independent learning, because in the absence of continuous contact with the teacher, they had to make a greater effort on their own to understand and to learn the concepts [42]. Students indicated that this aspect could be very useful as a complement in a face-to-face class, although not in the sense of above-mentioned complementary projects, but from the point of view of reinforcing what was explained in class. Additional exercises could supplement those carried out in class, indicating only the solution, without the development, and on a voluntary basis. Thus, students who wanted to could solve them and deepen their knowledge of the aspects that were explained [19]. This activity should be complemented with the availability of the teacher to resolve any doubt that might arise about these exercises.

*“[...] it’d be interesting, if we were provided with teaching material to look more deeply at the aspects that were explained [...] especially regarding problems [...].”* (Week 6). *“I consider that supplementary material that addresses the aspects discussed in class can be very useful [...].”* (Week 7). *“[...] I have always missed additional problems to solve on my own [...].”* (Week 8). *“[...] during this confinement I have seen that I can also learn by myself [...] the teacher should promote this behavior through exercises linked to the concepts explained in class, never through extension work whose topic is not related to what has been explained in class.”* (Week 9).

Finally, students stated that the support of their peers is also very relevant in their learning. According to the students’ comments, doubts are often solved among peers by asking each other. The use of a less technical language and the greater trust they have among themselves compared to the teacher favors this type of behavior [39]. Therefore, students affirmed that it would be interesting for the teacher to promote activities that favor this support among classmates so that those students who understand a certain concept better can explain it to the others. In addition, those sorts of activities could make classes more enjoyable and encourage students to attend class [5].

*“During online teaching I have missed the support of my classmates [...] for me it is very important since I usually ask them doubts [...].”* (Week 6). *“[...] I often ask my classmates things about the class [...] the teacher could favor activities aimed at this [...].”* (Week 7). *“[...] I think that going deeper into the concepts together with my classmates would help me understand everything better [...].”* (Week 7). *“I’ve learnt a lot from group projects in which everyone works, they should be more regular [...].”* (Week 8). *“[...] the teacher should think of activities in which the students work in groups [...] it favors learning if the group work is good and makes the classes more enjoyable.”* (Week 9).

**Figure 7:**
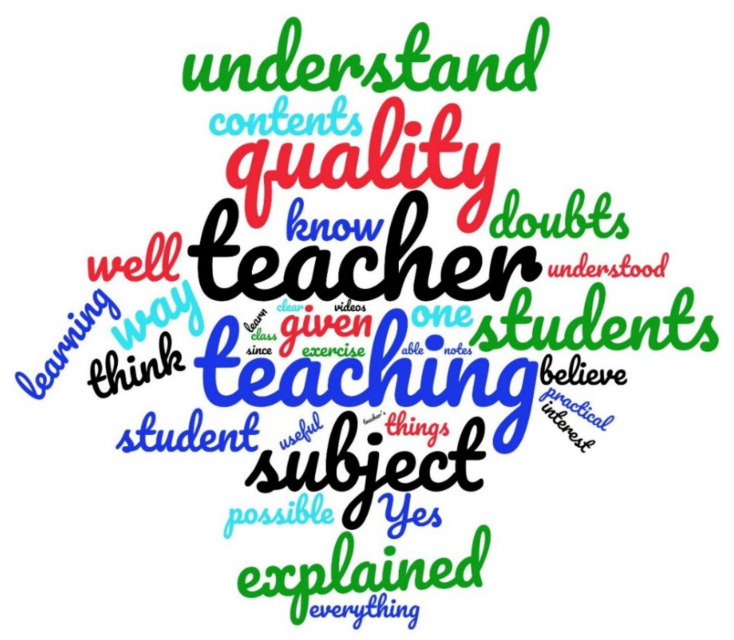
Word Cloud of question 5 for Week 9.

Figure 7 shows a Word Cloud based on word counting obtained from the students’ answers to question 5 of the survey in Week 9. This Word Cloud displays the importance of the term teacher. The teacher is the guide to the course and is in charge of implementing the seven aspects indicated by the students and commented upon in this section. Hence, the role of the teacher is fundamental to promoting student learning. Therefore, the correct work of the teacher in engineering courses is essential [5], as discussed in the following section.

## 4. Overall Discussion

**Figure 8:**
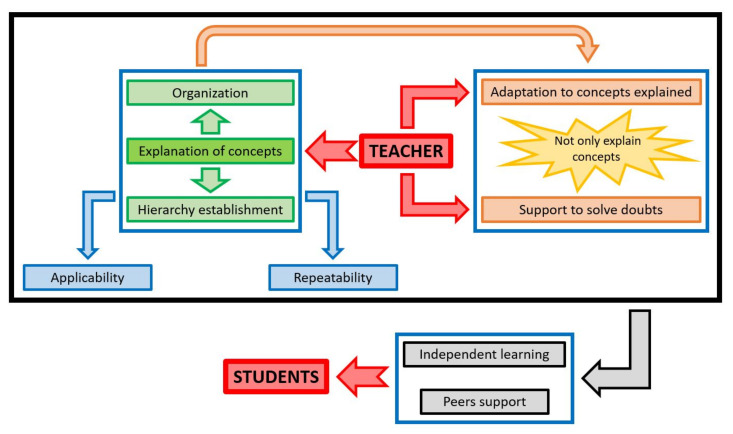
Conceptual diagram of elements needed for successful learning in engineering.

With the aim of offering an overview of all learning elements reported by the students, Figure 8 shows a conceptual map linking the seven aspects that the students highlighted as necessary for successful engineering learning: explanation, organization, and hierarchization of concepts; adaptation to the concepts as they were explained; teacher’s support; exemplification and applicability of concepts; repetitiveness of concepts; autonomy; and peer support. This figure is intended to show how these aspects should be related to each other when approaching an engineering course.

All engineering courses are characterized by a close link between theoretical and practical concepts, which implies that there must be an adequate bridge between both [9]. This task of simultaneously approaching both theoretical and practical concepts is usually performed by the teacher, so that in traditional engineering courses, the teacher explains the theoretical concepts and then applies them to the resolution of practical cases [14], which explains why in engineering teaching the role of the teacher is fundamental. Therefore, theory and practice cannot be approached separately in engineering courses [5], but rather, the teacher must find a way to link them and explain both types of concepts simultaneously.

This fundamental role of the teacher led students who participated in this study to organize engineering teaching around the figure of the teacher. First, they indicated that the teacher should explain the concepts following a logical scheme (organization), i.e., the concepts addressed later in the syllabus should be based on previously taught concepts. The explanation of concepts should not be done randomly. This practice is common and usual [43], but it must be complemented with an adequate hierarchization, so that the teacher should identify which are the key or the most important concepts of each topic that is addressed. It has been shown that this practice facilitates adequate organization of student learning, which leads to better overall learning on the course [44]. In engineering, where the range of concepts learned by students is very wide [4], hierarchizing the concepts can undoubtedly facilitate learning.

In this explanation of the concepts, the teacher should implement two practices that in the students’ own words facilitate their learning processes. On the one hand, key concepts should be systematically repeated throughout the course, in order to link the different aspects explained to each other and to help students to situate themselves within the great diversity of concepts addressed in engineering courses [45]. Moreover, the concepts should be adequately linked with real examples. The teacher’s experience and knowledge of the professional world are essential in engineering, since it is a profession with a direct application in the real world [8]. The teacher should explain the concepts based on real examples, as this practice allows an easier understanding of engineering concepts, as well as motivating students in their learning [29].

This explanation of the concepts, which should be organized, hierarchical, repetitive, and exemplified, results in the need for the teacher to provide teaching material and to request evaluation projects. In addition, students will pose doubts about the concepts that are explained. The teacher’s role, in relation to these two elements, again becomes fundamental [46]. First of all, both the notes and the projects requested from the students should be adapted to the concepts that have been explained. The notes should be adjusted to the practical concepts, if these are a fundamental element of the course; it is not enough to have notes that only address the theoretical aspects. The projects should be related to the concepts explained in the classroom, so that they are studied in greater depth. The students did not consider the supplementary work of any utility, as these topics were somewhat removed from the subject matter covered in class. Secondly, the teacher should offer continuous support to solve students’ doubts, including those referring to knowledge that is assumed to have been learnt on previous courses. The teacher’s availability should not be limited to the classroom [34], but the teacher should also be available both through tutorials and e-mail, a channel through which students currently ask most of their questions, even in face-to-face teaching [16].

All the aspects addressed so far result in the need for traditional teaching, but with major teacher involvement in different dimensions not generally considered in engineering courses. However, the students also highlighted elements typical of unconventional teaching methodologies: independent learning and peer support. Usually, the independent learning of students following engineering teaching has been limited to the completion of assignments/projects, but it should go beyond that. For example, additional voluntary exercises can be provided, so that students can solve them and go deeper into what they have been taught in class [47]. Another possibility is to let students make a first independent approach to both theoretical and practical concepts. To that end, the flipped classroom can allow students a certain understanding of the theoretical concepts prior to class [48], while cooperative work allows students to work in groups to solve exercises after a brief explanation of the theoretical concepts by the teacher [49]. The latter teaching methodology also favors peer support through working in groups and if adequate formative evaluation is conducted [12], although a simple classroom discussion among students can cover the support they demand [5]. The greater trust that any student has with their classmates rather than with the teacher, as well as the use of a less technical language among them, favors quick and efficient resolution of a large number of doubts [14]. Undoubtedly, these two aspects, autonomy and peer support, are the major shortcomings in current engineering teaching, although they are demanded by students. An increasing number of teaching methodologies that promote these aspects are being progressively applied in engineering teaching, but formal presentations remain the most common teaching practice [10], which does not allow students to learn as much as they could according to their own opinion. Both formal presentations and independent teaching methodologies are necessary, and teaching methodologies that promote them should therefore be implemented in engineering courses. Different studies have shown the validity for students’ learning of, for instance, project-based learning or cooperative work, which can replace the traditional classes performed for the resolution of exercises [5,41,50].

## 5. Conclusions

The results of an experimental study to determine the aspects that students considered fundamental to learn engineering have been reported in this paper. The lockdown caused by the COVID-19 pandemic and which resulted in a sudden and unexpected adaption of face-to-face teaching to an asynchronous online methodology provided a suitable setting for students to reflect on these issues. To that end, a survey was designed to encourage a critical analysis among students of both types of teaching, face-to-face and online, and to identify the advantages and disadvantages of each, and then to share their thoughts on engineering teaching to ensure their learning. Students were asked to answer this survey every week throughout the duration of the online teaching. The aim was to encourage students to deepen their reflections, as well as to identify the stage of reflective behavior of the students successfully, i.e., when they had accepted the change to online teaching (without complaints or resignation) and began to reflect on how it could be improved considering the face-to-face teaching they had traditionally received. All the conclusions indicated below are based on this stage of behavior, in which the survey was answered in a thoughtful and reflexive way.

Firstly, approximately 65% of students preferred face-to-face teaching, an aspect that was mainly motivated by the habit of the type of teaching they had traditionally received. However, between 90% and 95% of the students indicated that online teaching based on an asynchronous methodology using videos was adequate, finding three main advantages to this type of teaching: the speed at which the doubts were solved through e-mail exchanges, the flexibility to organize their own study program, and independent learning, so that they studied the aspects explained in the classroom effectively. Thus, students implicitly indicated that certain aspects of face-to-face teaching could be improved by incorporating elements of online teaching.

Based on this reflection, the students indicated that teaching in engineering should be based on an explanation of concepts that is not only organized, but also hierarchical, clearly identifying the key concepts of each topic addressed, which should be conveniently repeated throughout the course, and whose applicability in the professional world should also be clearly shown. The other two aspects requested by the students were that the teachers adapt the work requested and the preparation of the notes to the concepts addressed in the course, and that they provide support for the resolution of doubts, especially by e-mail. The students were therefore proposing a teaching scenario in which the teacher plays an essential role, not only in relation to the explanation of concepts, but also with respect to how these concepts should be explained and organized to ensure learning. It is not enough, therefore, to explain the concepts, but this explanation must be done in a particular way. This main role of the teacher highlighted by students was expected, and it is because of the close link between theoretical and practical concepts, common in any engineering course.

Within this framework, in which the teacher is the main figure, the activities should promote autonomy and peer support in learning, two elements not generally considered in engineering teaching. After experiencing asynchronous online teaching, the students themselves demanded the systematic incorporation of these two elements, which would not only make the teaching of the courses more enjoyable, but would also bring the students closer to the future professional world and show the students how to carry out continuous learning, which is necessary in engineering throughout the professional life. It is therefore necessary to incorporate teaching methodologies that favor these aspects and complement the explanation of the concepts by the teacher.

## Data Availability

The data presented in this study are available on request from the corresponding author. The data are not publicly available due to the need to maintain the participants’ anonymity.
